# Open and Distance Learning Programs for Nursing and Midwifery Education in East Africa: Protocol for a Scoping Review

**DOI:** 10.2196/17765

**Published:** 2021-01-11

**Authors:** Kahabi Isangula, Grace Edwards, Tumbwene Mwansisya, Columba Mbekenga, Eunice Pallangyo, Ahmed Sarki, Eunice Ndirangu-Mugo

**Affiliations:** 1 School of Nursing and Midwifery Aga Khan University Dar Es Salaam United Republic of Tanzania; 2 School of Nursing and Midwifery Aga Khan University Kampala Uganda; 3 School of Nursing and Midwifery Aga Khan University Nairobi Kenya

**Keywords:** open and distance, learning, health care, nurses, midwifery, health, East Africa

## Abstract

**Background:**

In the face of growing modernity and the coronavirus disease 2019 (COVID-19) pandemic, open and distance learning (ODL) is considered to play an important role in increasing access to education worldwide. There is a robust evidence base demonstrating its cost effectiveness in comparison with conventional class-based teaching; however, the transition to this new paradigm of learning for nursing and midwifery courses has been difficult in low-income countries. While there are notable efforts to increase internet and education access to health care professionals, not much is known about ODL for nurses and midwives in East African countries.

**Objective:**

The objective of this scoping review is to understand whether ODL programs for nursing and midwifery education exist, the drivers of their adoption, their implementation, the topics/courses covered, their acceptability, and their impacts in East African countries.

**Methods:**

The scoping review methodology employs the framework developed by Arksey and O’Malley. Using an exploratory approach, a two-stage screening process consisting of a title and abstract scan and a full-text review will be used to determine the eligibility of articles. To be included, articles must report on an existing ODL initiative for nurses and midwives in Uganda, Tanzania, and Kenya. All articles will be independently assessed for eligibility by pairs of reviewers, and all eligible articles will be abstracted and charted in duplicate using a standardized form.

**Results:**

Details of ODL for nursing and midwifery education initiatives and study outcomes will be summarized in a table. The extracted data will undergo exploratory descriptive analysis, and the results will be classified into learner and clinical outcomes.

**Conclusions:**

Evidence on ODL for nursing and midwifery education will inform the ongoing development and restructuring of health care professional education in East Africa amidst the COVID-19 pandemic.

**International Registered Report Identifier (IRRID):**

PRR1-10.2196/17765

## Introduction

In the face of modernity, the coronavirus disease 2019 (COVID-19) pandemic, and increased access to the internet, telecommunication, and related technologies, there is growing interest in open and distance learning (ODL) as a promising entry point to address the challenges of access to educational opportunities worldwide [1-5]. ODL entails a process that facilitates acquisition of knowledge and skills through open and flexible information and instruction mainly by using technology, with all or most of the teaching delivered by an individual who is at a distance from the learner [5,6]. In recent years, ODL has become a foundation for initial training for formal qualifications, in-service training for formal upgrading, and continuing in-service training in particular subjects and topics in many high-income countries and some low-income countries [5,6]. Through ODL, students in one part of the world/country are able to earn educational qualifications from institutions located in other parts of the world/country. Although it may not carry the full package of benefits enjoyed in traditional class-based teaching, a growing body of research continues to provide evidence of the effectiveness of ODL in both high- and low-income countries. Some studies have reported a similar impact between ODL and conventional class-based learning strategies [6,7]; however, a considerable number of studies report ODL as playing a role in increasing student enrollment and performance, and it is mostly preferred by students with work commitments as compared with face-to-face education [8-12].

The world continues to face challenges regarding human resources for health. Concerns of low production from health training institutions and consequently inadequate number of health care workers, as well as gaps in knowledge and skills are large particularly in sub-Saharan Africa [13-16]. The disconnect between the persistent challenge of inadequate and skilled health care workers versus current production under traditional teaching models calls for new teaching strategies for health care profession education to address the challenges regarding human resources for health. The ongoing COVID-19 pandemic has further strained the available human resources for health in Africa, creating a demand for new knowledge and skill sets among health care professionals for personal and patient protection [2,3,17,18]. Likewise, long-standing academic staff shortages in East Africa and recent campus closures due to COVID-19 have caused many universities in the region to consider new teaching delivery methods, with ODL becoming a preferred option [2,3,18]. Backed by the growing and robust evidence of increasing recognition and implementation of ODL across the world [8-12], there is a need for its integration into the formal teaching and learning model within the health care sector. However, transition to this new paradigm of learning for health care professional–related courses in low-income countries, particularly East Africa, remains difficult. The transition from traditional class-based learning to ODL is a challenge, as there are many policy, infrastructural, and structural barriers that training institutions and learners, who are used to face-to-face education, must navigate [19-25]. While efforts to increase internet and education access to health care workers are evident, not much is known about ODL for nursing and midwifery in East African countries. The objective of this scoping review is to understand whether ODL programs for nursing and midwifery education exist; the drivers of their adoption; their implementation; their acceptability; the topics/courses covered; and their impacts in Tanzania, Kenya, and Uganda.

## Methods

### Design and Framework

Various evidence synthesis approaches were considered for this review; however, the scoping review methodology was seen as the most appropriate, especially since the complex area of ODL in East Africa has not been reviewed comprehensively before. To the best of our knowledge, there has been no prior attempt to establish a baseline of knowledge regarding ODL initiatives for nursing and midwifery education in the study setting. Given this knowledge gap and that literature may be diffuse owing to the multidisciplinary nature of ODL implementation, a scoping review is ideal for taking stock of the volume and nature of the existing literature [26]. The use of a scoping review as a form of exploratory knowledge synthesis allows for the broad exploration of ODL to map key concepts, evidence types, and gaps in research in a defined field and to make use of a wide array of knowledge exhibited through empirical research and anecdotal accounts [26-30].

The scoping review methodology employs the framework developed by Arksey and O’Malley from the Centre for Reviews and Dissemination at the University of York [27]. The framework provides a methodological approach to carry out this type of review, embracing some recent developments [28-30]. Arksey and O’Malley’s framework proposes the following five stages that will be followed for this review: (1) identification of the research question to be addressed; (2) identification of studies relevant to the research question; (3) selection of studies to include in the review; (4) charting of information and data within the included studies; and (5) collating, summarizing, and reporting results of the review. An optional sixth stage involves consultation with stakeholders to ensure comprehensive inclusion of all relevant materials [27]. We will use this framework to guide our exploratory scoping review, and where necessary, we will develop more specific procedures to carry out the stages of the review process. The following sections describe the methods that will be followed in our scoping review.

### Identifying the Research Question

Arksey and O’Malley [27] recommended the consideration of all aspects of the research area to generate a breadth of coverage. Drawing on the expertise of our research team, current discussions on teaching and learning in East Africa (Uganda, Tanzania, and Kenya), and an initial scan of the literature, we defined our overriding research question as follows: *What is the extent of published evidence in East Africa relating to the existence of ODL programs for nursing and midwifery education and their drivers, implementation, topics covered, acceptability, and impacts?* The rationale behind this broad question was the lack of harmonized evidence about ODL practices for nursing and midwifery education in East Africa. By identifying the current educational initiatives, this review seeks to establish a foundational understanding of how ODL programs for nursing and midwifery education, if any, are implemented and glean the critical impact factors and recommendations of these experiences. Based on a combination of informal discussions and a preliminary review of published topics, we developed the following specific questions for our exploratory scoping review: (1) *What ODL interventions exist for nursing and midwifery training programs in East Africa?* (2) *What are the key drivers for adoption of ODL interventions for nursing and midwifery in East Africa?* (3) *What aspects of nursing and midwifery education are taught using the ODL strategy?* (4) *How are the ODL interventions for nursing and midwifery education delivered? *(5) *What are the existing policy and institutional frameworks that guide and promote quality of ODL interventions?* (6) *What is the documented impact of ODL interventions for nursing and midwifery education in East Africa?*

### Stakeholder Consultation

The optional stakeholder consultation phase is meant to be an ongoing interaction throughout the review process [27]. Thus, we feel it is important to initiate contact with stakeholders at the beginning of the review process. Early involvement of stakeholders will allow us to seek guidance regarding our research question, thus ensuring that the results are of broad interest among different stakeholder groups.

Stakeholders of interest represent fellow researchers, instructors, and decision makers involved in nursing and midwifery education in East Africa. Individual stakeholders will be identified through professional networks and contacted via email. We will brief them on our research question and focus areas, as well as approach to searching the literature, and solicit their feedback on our approach. Only their titles and/or positions will be used to ensure anonymity.

### Identifying Relevant Studies

To be comprehensive, Arksey and O’Malley recommended searching several literature sources, including electronic databases, reference lists of relevant literature, key journals (hand searching), existing networks, relevant organizations, and conferences [27]. For our scoping review, we will approach this in multiple steps. First, a search strategy will be co-developed by the research team in collaboration with an experienced librarian targeting different formal and informal health care profession databases that promote African content. The search strategy will consist of subject headings, keywords, and related terms for ODL, nursing, and midwifery education, and all East African countries. The search terms applied in one database will then be translated for use in the other databases. Once relevant material is selected from databases, we will search relevant websites, URLs, and reference lists of key studies to increase our capture of relevant material.

### Electronic Database Searching

We will enlist the services of a library scientist to conduct the electronic database search. The research team will devise a broad list of terms pertinent to ODL in nursing and midwifery education, including ODL initiatives, ODL implementation, and ODL benefits/impacts. These terms will be combined to create keywords that could be used to search electronic literature databases. Keywords will then be mapped to database thesaurus search terms, where available, and also searched as text word terms in all databases. The goal is to conduct a sensitive rather than specific search of the literature; thus, search terms are kept very broad, resulting in many irrelevant studies being eliminated at the study selection. All literature database searches will be limited to the English language and publication from inception to December 2020.

### Website Searching

Once relevant studies are selected from the literature database search, we will carry out a selective search of relevant websites. Through consultation with our stakeholders and members of the research team and colleagues, we will compile a list of relevant websites in East Africa to search. We will attempt to search websites in a systematic manner, allowing for some variation in search strategies in response to varied website structures. Once hand searching a website’s links is complete, we will use the website’s search engine to attempt to uncover additional materials. Once again, different types of search engines require different search tactics. For all websites, we will search the terms *open and distance*, *nursing and midwifery education*, *nursing education*, *midwifery education*, and *East Africa OR Tanzania OR Kenya OR Uganda*. For websites that are not specifically health care focused, we will add the word “health” to the specific term. A log of the website searches will be kept, and links to relevant pages will be saved.

### Other Literature Sources

In an attempt to be as comprehensive as possible in our search, we will also collect literature from reference lists of relevant articles, master’s and PhD theses, specific journal issues with related material, technical reports from health care organizations, and suggestions from colleagues.

### Selection of Relevant Studies

A two-stage screening process consisting of a title and abstract scan and a full-text review will be used to determine the eligibility of articles. Both stages will follow the same process, where every article will be independently reviewed in pairs and the results will be documented on the spreadsheet. At the end of each round, the ratings will be compared and resolved by two reviewers or a third reviewer, when consensus is not achieved. Any ambiguities regarding the eligibility of a citation (or article) will be flagged and discussed.

The citations will be assessed for relevance based on a title and abstract scan. To be relevant for full-text review, articles must report on an existing ODL initiative for nurses and midwives in Kenya, Uganda, and Tanzania. The full-text review form will ask reviewers to assess each article using the following questions: *Does the article describe/discuss the ODL initiative/intervention/program for nursing and midwifery education in Tanzania, Kenya, and Uganda?*

This review is inclusive of all types of literature, thus including commentary articles, technical reports, case studies, and empirical studies employing all types of methodologies (ie, qualitative, quantitative, and mixed methods) and study designs. Viewpoint articles on how ODL education programs should be implemented outside of the context of an existing program will be excluded.

The criteria will be piloted by the reviewers to refine and establish a common understanding of the inclusion criteria. About 10% of the selected citations from a single database will be independently reviewed by four reviewers to establish interrater reliability (IRR). The results of the review will be compared, and the IRR will be calculated. The threshold for IRR is set at an average Cohen kappa of 0.70, indicating substantial agreement [31,32]. The pilot will be run again if the threshold is not met. If met, the remaining articles will be divided and assigned to two sets of pairs for independent review. These adjustments to the inclusion-exclusion process are appropriate, as they provide the team with opportunities to become familiar with the data and to reduce workload [32-34]. Regardless of the IRR outcome, a meeting about the process will be held to compare the results, resolve disagreements, and troubleshoot the challenges that arise during the title-abstract review process.

### Charting of the Data

**Figure 1:**
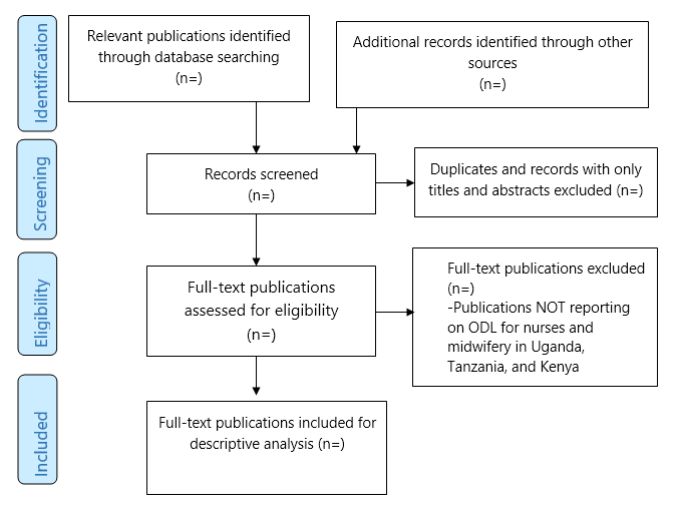
PRISMA flow diagram of the study. ODL: open and distance learning.

Arksey and O’Malley indicated that the charting process is multistaged, involving extraction of information from individual articles [27]. A PRISMA chart will be used to summarize the stages of the scoping review and the publications included (Figure 1). However, a detailed standardized charting form will be developed and used to categorize or “chart” the data. The high-level domains for the charting form consist of general citation information, health care profession area, level of reporting, country of origin, and key findings from the included articles; initiative details; and implementation factors. There will be a training session to trial the charting form and ensure there is a common understanding of the categories and how to use the form. The full-text reviewers will be asked if there are any additional variables emerging from the full-text review to consider for charting. The form will be piloted on 5 to 10 articles by the team. A final round of feedback on the form will be solicited prior to the charting process. The charting will also consist of independent charting by the reviewers and validation by the senior investigators. The charters will be encouraged to provide constant feedback on emerging themes not captured in the charting form. The form will be revised as required.

### Summation, Collation, and Synthesis

The purpose of this final stage of the scoping review is to provide a structure to the literature uncovered. The extracted data will first undergo a simple quantitative analysis using descriptive statistics (eg, frequencies) to provide numerical summaries of the education initiatives and article or study characteristics [27]. Multiple articles stemming from a single ODL initiative will be grouped and treated as a unit of analysis. The data will also undergo a “narrative review” or a descriptive analysis of the contextual or process-oriented data where all data will be thematically analyzed independently by two reviewers to identify emerging themes found within each of the subdomains (ODL availability, implementation, acceptability, and impacts). Focusing on the descriptive nature of the material in the charting phase will allow for the identification of additional categories and themes in the literature. The results will be compared and consolidated by consensus between the two reviewers. The resulting themes will be reviewed by content experts to ensure validity and credibility. The themes will be reported to highlight the similarities, patterns, differences, and outliers found in the literature.

## Results

The results from selected publications will undergo exploratory descriptive analysis and will be classified into learner and clinical outcomes [31-34]. The list of stakeholders consulted, electronic databases, and websites for the search will be tabulated. Details of ODL for nursing and midwifery education initiatives and study outcomes will be summarized in a table. The articles will not be assessed for quality as it is outside the scope of this review; however, details of the included articles (ie, article type and methodology) will be reported using a summary table to provide context for the maturity of the evidence.

## Discussion

The purpose of this review is to gain an understanding of the existence of ODL programs for nursing and midwifery education, the drivers of their adoption, the extent of their implementation, the courses covered, and their acceptability and impacts. The discussion of the findings will be contextualized based on previous studies and their implications for the ongoing development of ODL initiatives for nursing and midwifery education in East Africa (Uganda, Tanzania, and Kenya) in the face of the COVID-19 pandemic.

This scoping review is not without limitations. The review only focuses on nursing and midwifery education. This means ODL interventions on other aspects of health care professional education will be excluded. However, a snapshot of ODL interventions beyond the nursing and midwifery sphere will be offered to partly overcome this exclusion. The review only focuses on Tanzania, Kenya, and Uganda. While this is driven by the researchers’ institutional affiliation presence, further reviews may extend beyond these countries.

Research ethics approval is not required for this scoping review. This protocol reports a comprehensive, rigorous, and transparent methodology. This review contributes to the advancement of research on this subject and comments on the maturity of the body of literature by identifying gaps in knowledge and research. Through the publication of the results and their dissemination at relevant conferences, the findings of this review could guide the direction of future research and implementation of ODL initiatives for nursing and midwifery by health care institutions in East Africa amidst the COVID-19 pandemic. The results will also be presented at relevant national and international conferences and published in a peer-reviewed journal. The results of this review may inform the design of new initiatives and the policies that support them; moreover, future implementation teams can learn from the experience of others to avoid potential barriers and focus on enablers to increase the chances of success of their ODL programs (existing or new).

## Abbreviations

COVID-19: coronavirus disease 2019
IRR: interrater reliability
ODL: open and distance learning
